# Prey and predator overlap at the edge of a mesoscale eddy: fine-scale, in-situ distributions to inform our understanding of oceanographic processes

**DOI:** 10.1038/s41598-020-57879-x

**Published:** 2020-01-22

**Authors:** Moritz S. Schmid, Robert K. Cowen, Kelly Robinson, Jessica Y. Luo, Christian Briseño-Avena, Su Sponaugle

**Affiliations:** 1 Hatfield Marine Science Center, Oregon State University, Newport, OR 97365 USA; 2 Department of Biology, University of Louisiana at Lafayette, Lafayette, LA 70503 USA; 3 NOAA Geophysical Fluid Dynamics Laboratory, Princeton, NJ 08540 USA; 4 Department of Environmental and Ocean Sciences, University of San Diego, San Diego, CA 92122 USA; 5 Department of Integrative Biology, Hatfield Marine Science Center, Oregon State University, Newport, OR 97365 USA

**Keywords:** Marine biology, Image processing, Machine learning, Optical imaging, Biooceanography

## Abstract

Eddies can enhance primary as well as secondary production, creating a diverse meso- and sub-mesoscale seascape at the eddy front which can affect the aggregation of plankton and particles. Due to the coarse resolution provided by sampling with plankton nets, our knowledge of plankton distributions at these edges is limited. We used a towed, undulating underwater imaging system to investigate the physical and biological drivers of zoo- and ichthyoplankton aggregations at the edge of a decaying mesoscale eddy (ME) in the Straits of Florida. Using a sparse Convolutional Neural Network we identified 132 million images of plankton. Larval fish and *Oithona* spp. copepod concentrations were significantly higher in the eddy water mass, compared to the Florida Current water mass, only four days before the ME's dissipation. Larval fish and *Oithona* distributions were tightly coupled, indicating potential predator-prey interactions. Larval fishes are known predators of *Oithona*, however, Random Forests models showed that *Oithona* spp. and larval fish concentrations were primarily driven by variables signifying the physical footprint of the ME, such as current speed and direction. These results suggest that eddy-related advection leads to largely passive overlap between predator and prey, a positive, energy-efficient outcome for predators at the expense of prey.

## Introduction

Eddies are ubiquitous features of the ocean, turning mechanical energy into trophic energy^1^. The footprint of a mesoscale eddy can extend 100–300 km in diameter and can last for several weeks to months^2^. Through their upwelling effect, cyclonic mesoscale eddies (MEs) have been shown to enhance primary^3,4^ and secondary production^5–7^. This enhanced productivity may increase growth^8^ and survival^9^ of larval fishes, which normally experience up to 99% mortality due to starvation and predation^10^. Eddies may also physically retain larval fishes^11^, leading to higher larval fish concentrations inside eddies, relative to outside ambient waters, and are considered effective vectors for the transport of zoo-, and ichthyoplankton^12–14^. As such, mesoscale eddies play an important role in the connectivity of holo-and meroplankton populations^15^.

Eddy divergence and convergence patterns in the ocean lead to a cascading flow of energy from large to small scales^16^, with turbulent frictional coupling inducing smaller anti-cyclonic eddies at the periphery of larger cyclonic eddies thereby creating a feature- and energy-rich seascape^17^. Upwelling occurs in the centre of cyclonic MEs during their spin-up phase (termed a ‘forced’ eddy), but during the decay/spin-down phase (termed a ‘free’ eddy), this switches to downwelling at the core with upwelling occurring at the eddy edge^1,18^. In both instances, due to its frontal character, the eddy edge is an important feature for predator-prey interactions^1^. Less motile prey are often passively aggregated at the eddy edge and can be exploited by higher trophic levels and top predators including frigate birds and cetaceans^19–21^. The same physical eddy characteristics can lead to the entrainment and transport of larval fishes, facilitating their settlement to nearshore habitats^9,22^.

Despite the ecological importance of these features, insight into the distribution of plankton both within and at the edge of MEs is limited due to a lack of sufficient fine-scale vertical and horizontal resolution to adequately describe these distributions^7,23^. These limitations make it difficult to discern eddy edge/frontal effects on plankton or to further study the entrainment of plankton into eddies. A better understanding of zoo- and ichthyoplankton distributions around the edges of MEs, including the processes driving these distributions, would not only further our basic understanding of these ubiquitous features, but increase in the accuracy of biophysical transport models. Such biophysical models are used to estimate dispersal and population connectivity^15^, and ultimately, contribute to the spatial management of reef fish stocks^24,25^.

Gaps in our knowledge of plankton distributions in eddies stem from the limitations of traditional plankton sampling. For instance, net-based sampling enables only coarse horizontal and vertical resolution of plankton distributions^26–28^ and is thus inadequate for sampling across the edge of a ME where substantial physical changes occur over small spatial gradients^1,29^. Furthermore, since most zoo- and ichthyoplankton sampling is conducted with nets, there is often a mismatch between these samples and the finer spatial (<1 m) and temporal resolution (<1 s) at which physical properties of seawater can be sampled^26,27^.

A closer match between the scales of distribution of larval fishes and their patchy prey fields can be achieved using new sampling techniques such as underwater imaging, where data are collected on the scale of the individual and can reveal intriguing intra-genus spatial distributions^30^. Underwater imaging has come a long way since the beginnings of silhouette plankton photography^31^ and several imaging systems are in existence today (e.g., VPR^32^, ISIIS^27^, UVP5^33^, and LOKI^34^). These imaging systems were all designed to optimally sample different focal taxa. The VPR is towed behind a ship to investigate plankton ranging from diatoms and dinoflagellates to mesozooplankton^35^, while the UVP5 measures vertical profiles targeting small heterotrophs, particulate organic matter, and copepods, and is mounted on the CTD rosette^36^. LOKI samples vertical profiles of copepods and can image internal lipid reserves^37^. To quantify larval fish abundances and other meso-zooplankters, ISIIS samples the largest volume (150–185 L^−s^) of all existing imaging systems^27,38^.

Despite the advantages of *in-situ* imaging systems, their usage remains limited. Imaging gear is expensive relative to traditional plankton nets, and they often collect vast amounts of data (gigabyte to 10's of terabytes per cruise, translating into millions to billions of plankton images), which either have to be analysed and classified manually^29,39^, or automated using machine and deep learning^40–42^. Only recently have algorithmic approaches become sufficiently accurate, and graphics processing units (GPUs) powerful enough, to tackle this task. The state of the art in the automated identification of plankton specimens from underwater images utilizes convolutional neural networks (CNNs^42–44^).

Combining *in-situ* underwater imaging with a deep learning approach for the automated identification of plankton images, we investigated the physical-biological processes shaping zoo- and ichthyoplankton distributions in a decaying ME in the Florida Straits. Using the towed *In-situ* Ichthyoplankton Imaging System (ISIIS), fine-scale vertical sampling of the transition from eddy interior through the eddy edge was possible, providing unprecedented insight into the distributions of larval fishes as well as their potential prey. We hypothesized that the ME would structure zoo- and ichthyoplankton distributions, and that the distributions of small mesozooplankton taxa such as copepods would be driven to a large extent by eddy-induced advection while larger ichthyoplankton would be responsive to the presence of prey and predator taxa. Further, the eddy's effect on plankton distributions was predicted to weaken as time progressed towards the dissipation of the eddy.

## Results

### Environmental setting

**Figure 1:**
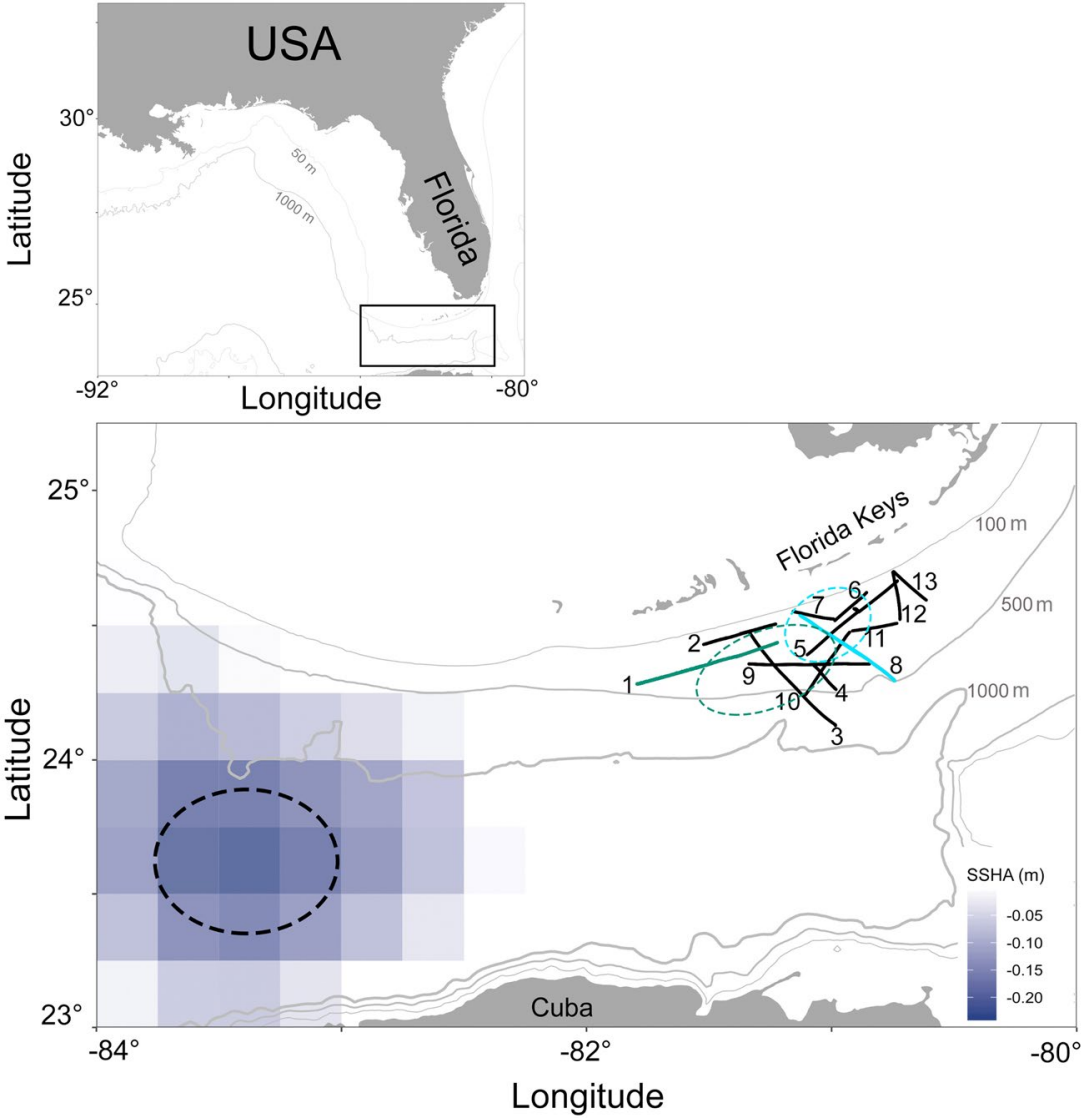
Sampling carried out to study the mesoscale eddy (June 10-16, 2015). Solid black lines and numbering delineates the transects sampled with the *In-situ* Ichthyoplankton Imaging System (ISIIS; see Supplementary Table S1 for details on transects). The dotted black ellipse marks the approximate position of the eddy on May 13, 2015, when it exerted the deepest sea surface height depression (indicated by blue tiles). Transect 1 (green solid line) and transect 8 (turquoise solid line) were subsequently analysed in more detail; the dotted ellipses in green and turquoise reflect the eddy positions on June 10 and 14, when transects 1 and 8, respectively, were sampled. This figure was made in R^81^.

Satellite Sea Surface Height Anomaly (SSHA) data revealed that the sampled ME formed on May 3, 2015, and was strongest (highest SSH depression) on May 13 (Fig. 1). Our sampling occurred later in the eddy's life cycle (June 10-16, Fig. 1), which was followed closely by the eddy's dissipation on June 18, 2015.

**Figure 2:**
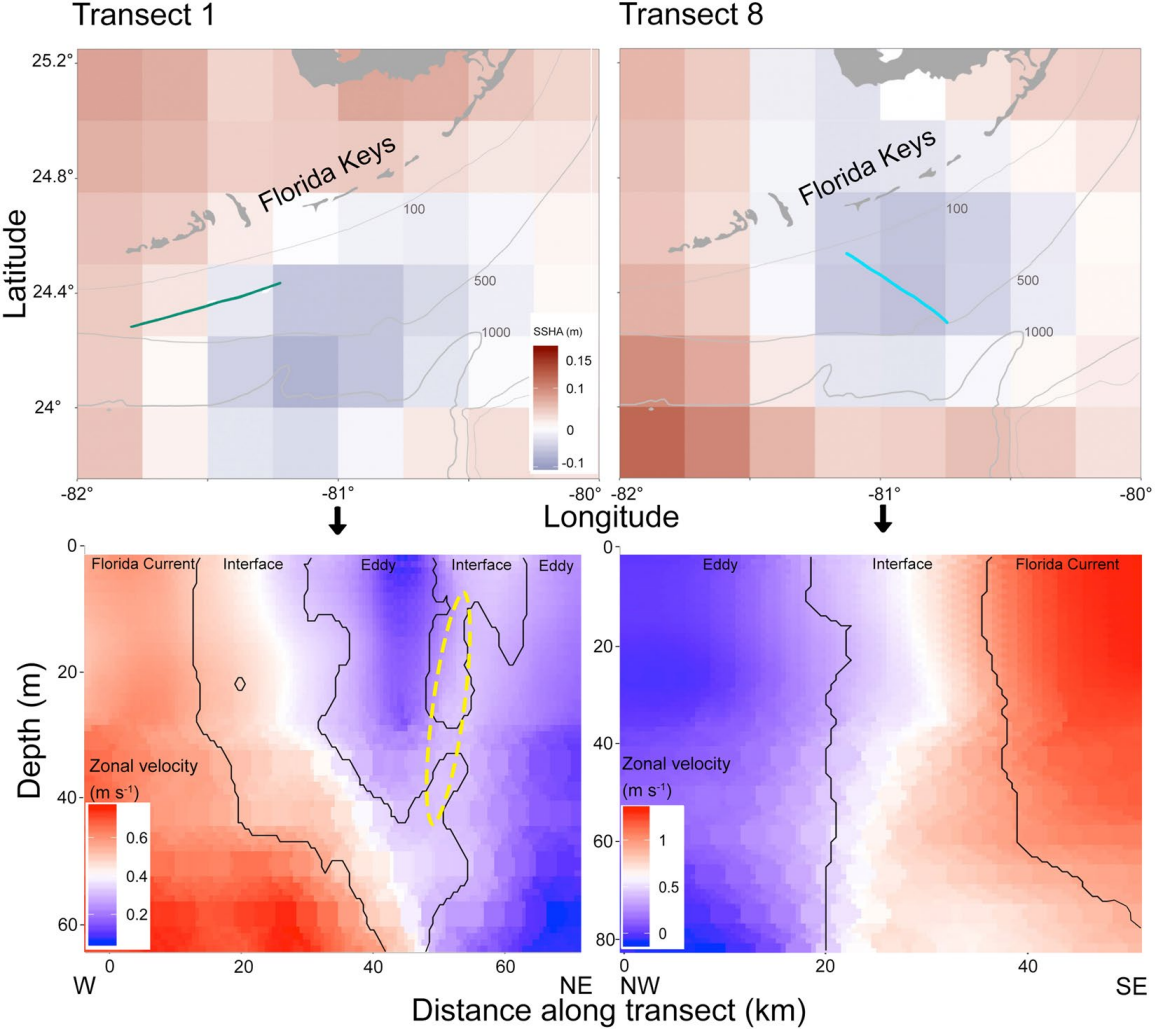
Transects 1 and 8 in relation to satellite sea surface height-, and zonal velocities from Acoustic Doppler Current Profiler data. Top panels show transect 1 (solid green line) and transect 8 (solid turquoise line) sampled with the *In-situ* Ichthyoplankton Imaging System (ISIIS), overlain on the sea surface height anomaly. Bathymetry contours are in light grey. Bottom panels show zonal velocities from the ship-board Acoustic Doppler Current Profiler (ADCP). Distance along transect begins in both cases at the westernmost end of the transect. Overlaid on top of the zonal velocities are the contours of the water masses as quantified by k-means clustering (see Methods), as well as the interface water filament (yellow ellipse). Transect 1 was sampled on 6/10/2015, and transect 8 on 6/14/2015. For a quiver plot of current speed and direction, please see Supplementary Fig. S4. This figure was made in R^81^.

The SSHA, together with zonal (Fig. 2) and meridional (Supplementary Fig. S1) velocities derived from a ship-born Acoustic Doppler Current Profiler (ADCP) showed that the progression from Florida Current (FC) water to the inside of the eddy, crossing the eddy edge, was clearly sampled on transects 1 (6/10/15) and 8 (6/14/15; Fig. 2). Current direction and speed data for the other sampled transects did not show an eddy edge allowing to differentiate eddy- and FC waters, and these transects were not further analysed. On transect 1, extending West to Northeast, the ISIIS imager was towed in an undulating fashion from Florida Current (FC) water into the eddy. On transect 8, extending Northwest to Southeast, ISIIS crossed from the eddy into FC water.

In order to distinguish water masses along the transects, we conducted a k-means unsupervised clustering analysis of the ADCP data (see Methods). The results revealed three significant current clusters (Fig. 2), explaining 99.74% of the variance on transect 1 and 99.87% of the variance on transect 8. On both transects the cluster with the slowest current speed, the smallest zonal (*u*), and the smallest meridional (*v*) vectors was indicative of the cyclonic ME, while the fastest speed and the largest *u* and *v* represented the FC^45,46^ (Supplementary Table S1). The cluster with intermediate speed, *u* and *v* was identified as *interface water* (IF), signifying the eddy edge. On transect 1, the IF formed a filament reaching into the eddy water mass (Fig. 2; yellow dashed ellipse).

Chlorophyll-*a* reached 0.6 µg l^−1^ in short, localized sections of transect 1 at around 60 m depth, but it was otherwise <0.3 µg l^−1^ (Supplementary Fig. S2). Coincident with enhanced phytoplankton biomass were the highest dissolved oxygen values measured on transect 1 (4.8 ml l^−1^). Temperature and salinity anomalies were strongest between 30 and 60 km along the transect, which was also visible in the shoaling of the isopycnals in this same section of the transect (Supplementary Fig. S2). On transect 8, chl *a* reached a localized maximum of 1.6 µg l^−1^ at ~ 15 km along the transect and between 60–80 m depth (Supplementary Fig. S3). Dissolved oxygen followed the chl *a* distribution closely, ranging from ~ 3.8 ml l^−1^ to 5.2 ml l^−1^. The temperature and salinity anomalies were most pronounced between 12 and 30 km along transect 8, which was also reflected in the shoaling of the isopycnals (Supplementary Fig. S3).

### Sparse convolutional neural network (sCNN)

**Figure 3:**
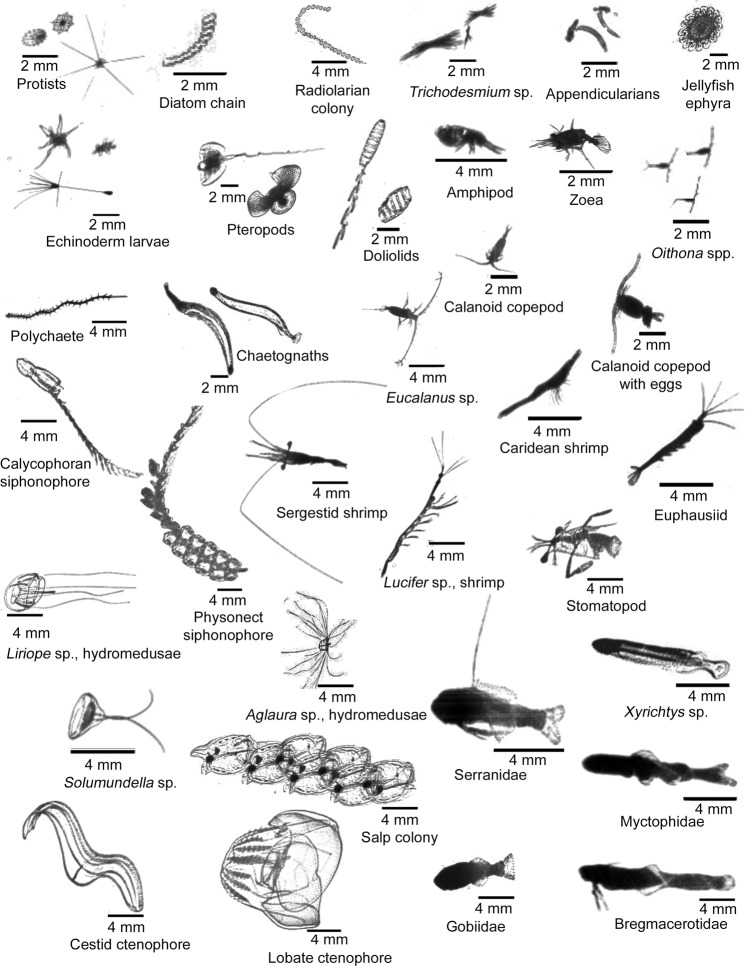
Plankton imaged with the *In-situ* Ichthyoplankton Imaging System. A representative collection of phyto-, zoo-, and ichthyoplankton images taken during this study in the Straits of Florida.

ISIIS recordings yielded 132 million image segments, including representatives of phytoplankton, *Oithona* spp. copepods, shrimp, gelatinous zooplankton, and ichthyoplankton (Fig. 3). After applying filtering thresholds and mapping the 124 original sCNN classes to 40 broader groups for further ecological analyses, the weighted average precision (number of true positives/number of true and false positives) was 96% across all groups, while the weighted average recall (number of true positives/number of true positives and false negatives) was 91% (Supplementary Table S2). Weighted mean F1 (i.e., the harmonic mean of precision and recall) was 93% (Supplementary Table S2). Precision for larval fishes, a rather rare but key group, was high (88.2%); however, recall (41.2%) was somewhat low, yielding a F1 score of 56.2%. Diatoms, appendicularians, chaetognaths, and *Oithona* spp. copepods are examples of organisms that all had F1 values> 90% (Supplementary Table S2). A correction factor^38,42^ (precision divided by recall) for each group was applied to calculate the final (i.e., corrected) concentrations of individuals m^−3^ (Supplementary Table S2). For example, the correction factor for larval fishes was 2.14.

### Plankton and particle concentrations along the transects

Average corrected concentrations of larval fishes were 5.6 and 2.3 ind. m^−3^ on transects 1 and 8, respectively. The most abundant copepod on transect 1 was *Oithona* spp. with a concentration of 60.8 ind. m^−3^, while on transect 8, ‘calanoid copepods’ were more abundant at 31.3 ind. m^−3^ than *Oithona* spp. with 15 ind. m^−3^. The cyanobacterium *Trichodesmium* was the most abundant organism on transects 1 and 8, with 140 and 85.3 ind. m^−3^, respectively, followed by appendicularians with 74.6 and 69.8 ind. m^−3^, respectively. The most abundant gelatinous zooplankton were chaetognaths at 21 and 18.8 ind. m^−3^ on transects 1 and 8, respectively. See Supplementary Table S3 for concentrations of all taxa.

Concentrations of larval fishes, as well as *Oithona* spp., differed significantly between the different water masses within the two transects. Concentrations of both taxa were significantly higher in the eddy (ED) water mass, compared to interface (IF) and Florida Current (FC) water; except the comparison of larval fishes between IF and FC water of transect 8 (ANOVA, Tukey; Supplementary Table S4).

**Figure 4:**
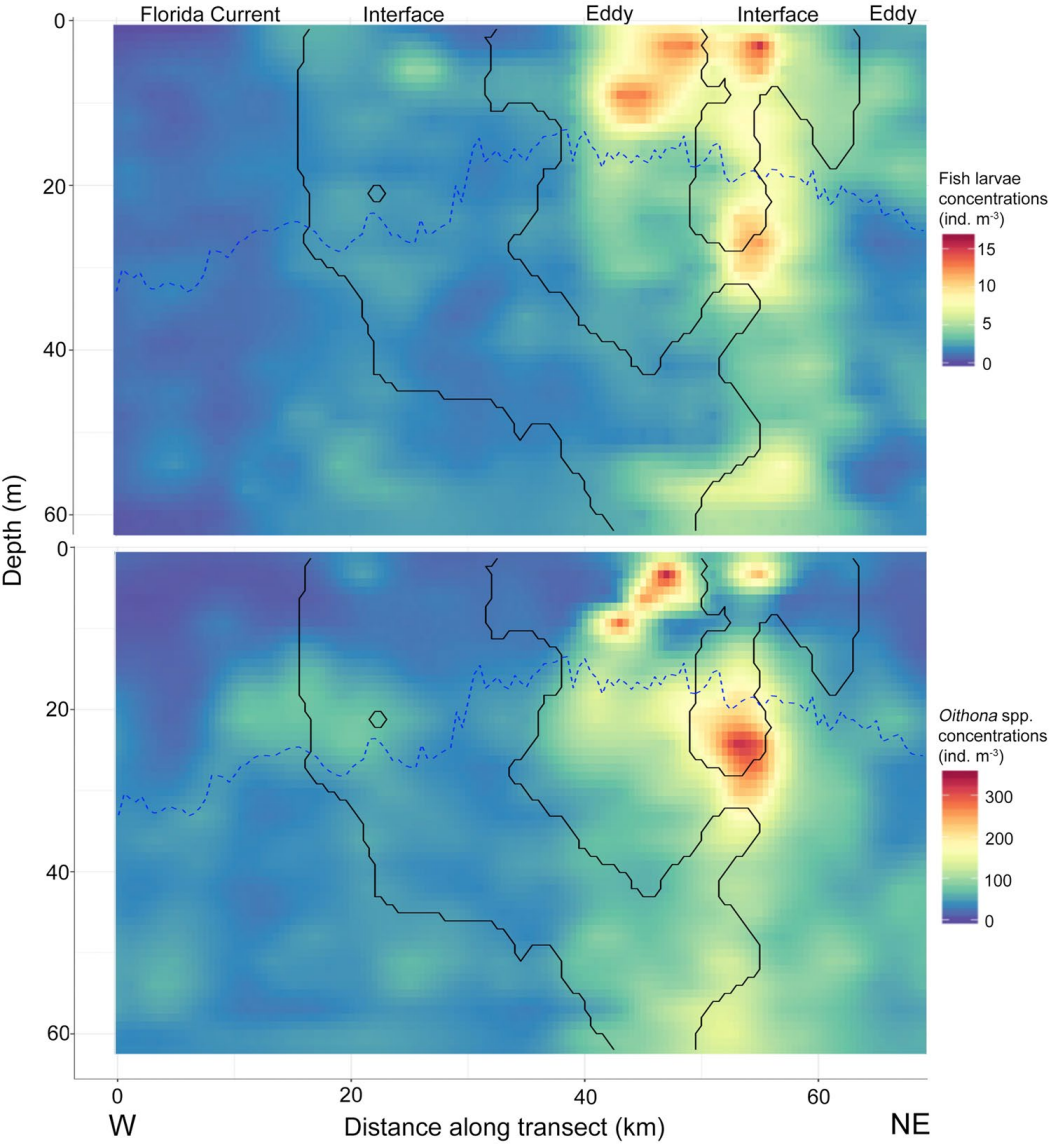
Concentrations of larval fishes (top panel) and *Oithona* spp. (bottom panel) along transect 1. Sampling was carried out from the West (W) to the Northeast (NE). Solid black lines indicate the boundaries between water masses as derived from k-means clustering. Water masses are labelled at the top of the panel. The blue dotted line indicates the mixed layer depth.

**Figure 5:**
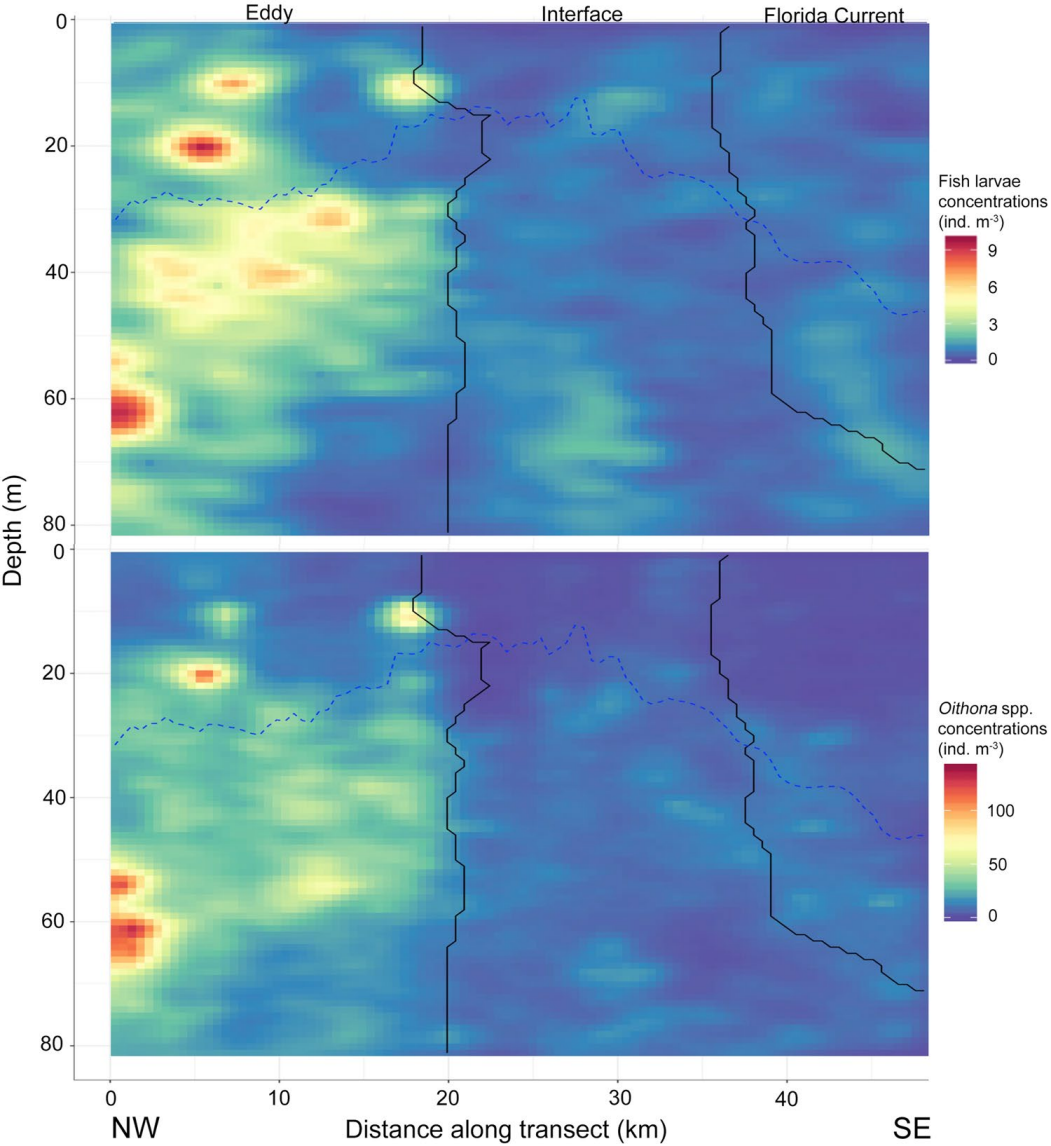
Concentrations of larval fishes (top panel) and *Oithona* spp. (bottom panel) along transect 8. Sampling was carried out from the Northwest (NW) to the Southeast (SE). Solid black lines indicate the boundaries between water masses as derived from k-means clustering. Water masses are labelled at the top of the panel. The blue dotted line indicates the mixed layer depth.

Fine-scale distributions of *Oithona* spp. along transect 1 and 8 overlapped spatially with the distributions of larval fishes, with the highest concentrations inside the eddy and interface water masses (Figs. 4, 5). On transect 8, larval fishes and *Oithona* spp. were more confined to only ED water than on transect 1 (Fig. 5). The mixed layer depth (MLD) shoaled on both transects in the sections pertaining to the interface water, while it deepened going further into the eddy. In both cases though, the MLD was deepest in the FC (Figs. 4, 5).

### Random Forests models

**Figure 6:**
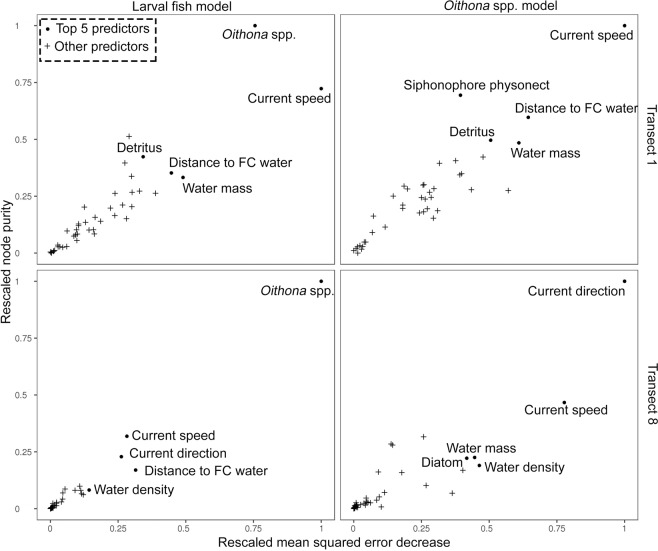
Explanatory variables used in the Random Forests models, ordered by their predictive importance. Left panels show the variables most important in the larval fish models of transects 1 and 8, while right panels show the most important variables in the two *Oithona* spp. models. Only the top five most important variables were labelled and further explored. The y-axis indicates the increase in node purity (an indicator for how well nodes in the decision trees split the data, see Methods section for more information) of the model when a certain explanatory variable was added, while the x-axis indicates the degree to which adding a certain explanatory variable reduced the mean squared error of the model.

The Random Forests model predicting larval fish concentrations on transect 1 (explaining 98.9% of variance) indicated that *Oithona* spp. concentrations and current speed were the most important variables in the model based on their effect on the decrease of Mean Squared Error (MSE) and increase in node purity (Fig. 6, see Table 1 for full list of variables). The next most important explanatory variables, in decreasing order, were: distance to FC water, water mass as derived from the k-means clusters (see Methods), and detritus concentrations. On transect 8, *Oithona* spp. concentration was the most important predictor of larval fish concentrations, followed in decreasing order of importance by: current speed, current direction, distance to FC water, and water density (Fig. 6). This Random Forests model explained 93% of total variance. Note that the explanatory variables for all Random Forests models included fine-scale concentration data for 34 taxa, including, for instance, calanoid copepods, and appendicularians (see Methods, Table 1).

**Table 1:** Explanatory variables used in the four Random Forests models.

No	Explanatory variable	Origin of data	No	Explanatory variable	Origin of data
1	Appendicularians	Concentrations from ISIIS imagery	23	Protists	“
2	Chaetognaths	″	24	Acantharia protists	″
3	Calanoid copepods	″	25	Pteropods	″
4	*Copilia sp*. copepods	″	26	Radiolarians	″
5	*Oithona sp*. copepods	″ *	27	Salps	″
6	*Oithona* sp. with eggs	“	28	Decapods	″
7	Other copepods	″	29	Euphausiids	″
8	Other crustaceans	″	30	Other shrimp	″
9	Cestid ctenophores	″	31	Calycophoran siphonophores	″
10	Cydippid ctenophores	″	32	Other siphonophores	″
11	Detritus	″	33	Physonect siphonophores	″
12	Diatoms	″	34	Trichodesmium	″
13	Doliolids	″	35	Current direction	ADCP derived
14	Echinoderm larvae, sea star	″	36	Current speed	″
15	Echinoderm larvae, sea urchin	″	37	Distance to Florida Current water	″
16	Fecal pellets	“	38	Water mass category (k-means)	″
17	Heteropods	″	39	Chlorophyll *a*	ISIIS sensors
18	Hydromedusae	″	40	Dissolved oxygen	″
19	Narcomedusae	″	41	Water density	″
20	Other jellyfish	″			
21	Pelagic tunicates	″			
22	Polychaetes	″			

Variables stem from three different datasets. Organismal data derived from *In-situ* Ichthyoplankton Imaging System (ISIIS) imagery (numbers 1–34, all measured in ind. m^−3^), variables derived from an Acoustic Doppler Current Profiler (ADCP; numbers 35–38), and *in-situ* environmental data from ISIIS sensors (numbers 39–41). *only used in larval fish models.

Since *Oithona* spp. concentrations were of such high importance to larval fish concentrations, subsequent Random Forests models were trained to differentiate the drivers of *Oithona* spp. concentrations. *Oithona* spp. concentrations on transect 1 were best explained by current speed, distance to FC water, physonect siphonophore concentrations, water mass, and detritus concentrations. On transect 8, the most important variables predicting *Oithona* spp. concentrations were the direction of the current, followed by current speed, water mass, water density, and diatom concentrations. The *Oithona* spp. Random Forests models on transects 1 and 8 explained 97.7% and 92.1% of the models’ variance, respectively.

### Accumulated Local Effects (ALE) on larval fish concentrations

**Figure 7:**
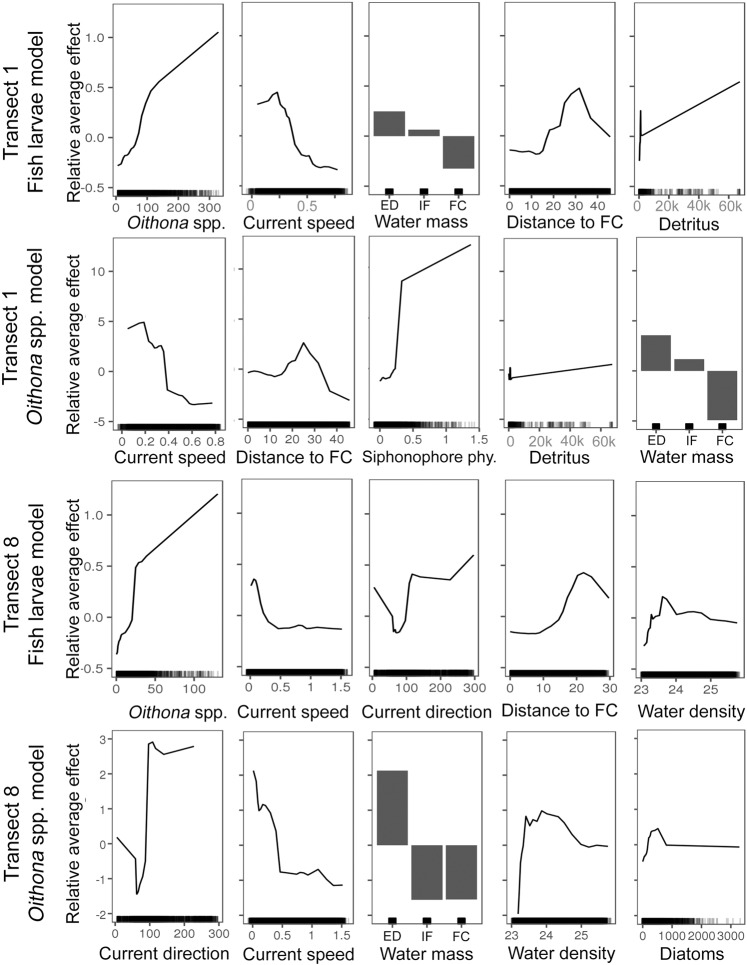
Accumulated Local Effects (ALE) exhibited by the explanatory variables in the Random Forests models on the respective predictor variables. Figures are shown for the top five explanatory variables of each of the four Random Forests models predicting larval fish and *Oithona* spp. concentrations. Each ALE plot shows if a value of a predictor leads to a below model-average (model average is indicated by the zero on the y axis) or above model-average prediction. Panels are ordered according to their importance in the Random Forests models (see Fig. 4), in descending order, from left to right. All plots within a model (panels found within a row) are centred on zero and can be compared with each other. Water mass categories: FC = Florida Current water, IF = interface water, and ED = eddy water. Siphonophore phy. = Physonect siphonophores.

Larval fish concentrations appeared to be substantially affected by distributions of *Oithona* spp.: higher *Oithona* spp. concentrations were associated with higher larval fish concentrations (Figs. 6, 7).

*Transect 1 –* Predictions of above model average larval fish concentrations occurred when *Oithona* spp. concentrations reached 50 ind. m^−3^, and peaked at 1 ind. m^−3^ more larval fishes than average, when there were 325 ind. m^−3^ of *Oithona* (Fig. 7). The slowest current speed (Supplementary Fig. S4) had the strongest positive effect on mean model predictions, while speeds> 0.25 m s^−1^ led to a sharp decrease in predicted larval fish concentrations. Presence of the FC water mass resulted in an average decrease of 0.3 ind. m^−3^ larval fish below model average, while interface water (IF) and eddy water (ED) increased the larval fish concentrations by an average of 0.1 and 0.25 ind. m^−3^. Predicted larval fish concentrations peaked at 0.5 ind. m^−3^ above the model average when individuals were about 30 km away from FC water, while within 20 km of the FC water edge average concentrations were below the model average (see Methods for how distance to the FC water mass was calculated). Further, larval fish concentrations increased steadily with the concentration of detritus in the water column, peaking at around 0.55 ind. m^−3^ more larval fishes than model average at detritus concentrations of about 60,000 particles m^−3^.

*Transect 8 –* Larval fish concentrations were predicted to rise above the model average when *Oithona* spp. concentrations reached 25 ind. m^−3^, and sharply increased until reaching 1.3 ind. m^−3^ above model average at *Oithona* concentrations of 130 ind. m^−3^ (Fig. 7). The slowest current speeds, between 0.05 and 0.25 m s^−1^, led to above model average larval fish concentrations, while speeds faster than 0.25 m s^−1^ generally led to below average larval fish concentrations. The direction of current flow was important for model predictions, with northward flowing water yielding the highest larval fish concentrations of around 0.65 ind. m^−3^ above model average. Furthermore, predicted larval fish concentrations were highest when larvae were at a distance of 22 km from FC water, while density influenced predictions in such a way that a density σ_t_ of 23.7 led to the highest mean model predictions of about 0.25 ind. m^−3^ above model average.

### Accumulated Local Effects (ALE) on *Oithona* spp. concentrations

While *Oithona* spp. concentrations were the most important explanatory variables in larval fish models on both transects, *Oithona* spp. concentrations themselves were mostly driven by abiotic variables such as current flow direction and speed (Figs. 6, 7).

*Transect 1 – Oithona* spp. concentrations were highest at the lowest current speeds: concentrations dropped substantially at current speeds above 0.35 m s^−1^ (Fig. 7). *Oithona* concentrations peaked at 3.5 ind. m^−3^ above model average when individuals were ~ 25 km away from FC water, while within 18 km of the FC water edge concentrations were below the model average. Mean model predictions of *Oithona* spp. concentrations were also above model average when the concentration of physonect siphonophores reached 0.25 ind. m^−3^ or more, and when the concentration of detritus particles reached 30,000 m^−3^. Presence of the FC water mass resulted in an average decrease of 4.95 ind. m^−3^ of *Oithona* below model average, while interface water (IF) and eddy water (ED) led to *Oithona* concentrations that were 1.5 and 4 ind. m^−3^ higher than model average, respectively.

*Transect 8 –* When water parcels had an eastward direction of flow, *Oithona* concentrations were 1.4 ind. m^−3^ below model average, while southward, southwestward, and northward flow led to concentrations up to 3 ind. m^−3^ above model average (Fig. 7, Supplementary Fig. S4). Current speeds below 0.4 m s^−1^ were associated with *Oithona* concentrations <2 ind. m^−3^ *Oithona* higher than the model average. *Oithona* concentrations in ED water were> 2 ind. m^−3^ higher than the model average, while IF and FC water lead to *Oithona* concentrations 1.5 ind. m^−3^ below model average. *Oithona* concentrations at water densities <23.3 were up to 2 ind. m^−3^ below the model average, while densities between 23.3 and 25 lead to higher than average predicted concentrations (Fig. 7).

To discriminate whether the high importance of *Oithona* spp. concentrations in the larval fish models was due to the underlying physical properties of the eddy driving the *Oithona* spp. models, or from a more active predator-prey interaction, we conducted a third set of Random Forests models, predicting larval fish concentrations without *Oithona* spp. as an explanatory variable. On transect 1, excluding *Oithona* spp. as a predictor from the larval fish model led to an explained variance of 98.6%, while removing *Oithona* spp. from the transect 8 model led to a reduction of explained variance to 91%. Thus, removing *Oithona* spp. as a predictor increased the variance by 0.3% on transect 1, and 2% on transect 8. To ensure comparability, all Random Forests models were based on the same settings (see Methods).

## Discussion

Use of the *In-situ* Ichthyoplankton Imaging System (ISIIS) and a sparse Convolutional Neural Network (sCNN**)** to automate the classification of 132 million plankton images enabled us to investigate the effects of a transient mesoscale eddy (ME) and its edge on the distribution of larval fishes and their *Oithona* spp. copepod prey^47,48^.

Consistent with the signature of a free, decaying cyclonic eddy^1,18^, we detected upwelling at the edge of the ME as indicated by the shoaling of the pycnocline between 30 km and 60 km along transect 1, and between 12 km and 30 km along transect 8. This area, termed interface water (IF) in our analyses and occurring between the eddy water (ED) and the Florida Current water (FC), delineates the eddy edge. This was further supported by the mixed layer depth (MLD), which was shallowest at the eddy edge, and deepest in the FC. When a forced cyclonic eddy turns into a free cyclonic eddy, surface waters converge instead of diverge, changing the eddy interior from upwelling to downwelling^1,18^. An effect of this downwelling is evident in the pycnocline of our ME which deepened as IF water transitioned into ED water, and the MLD, which was deeper further inside the eddy than compared to the edge. While a forced eddy is energetic due to the applied torque to initiate spinning, a free eddy is typically less energetic, with the primary torque coming only from the eddy itself, and strong opposing frictional torques slowing it down^1,17^, ultimately leading to the dissipation of the eddy's energy. Consistent with these energetics, shoaling of the pycnocline on both transects was rather gentle, likely related to the ME being just four days from total dissipation.

The velocity of FC water encountered in this study is well within the reported range, depending substantially on FC meandering, bathymetry, and distance to shore^49^. FC velocity can also vary on small spatial and temporal scales^49^, as was demonstrated here as well (0.8 m s^−1^ and 1.6 m s^−1^ on transects 1 and 8, respectively). Contrary to fast FC water, the current speed inside cyclonic MEs is much slower than in surrounding FC water^45,46^. Apart from these *in-situ* measurements from the ADCP, we also used satellite-derived measurements of the sea surface height anomaly (SSHA) to get a large-scale overview of the sampling area. While SSHA satellite data aligned well with transect 1 ADCP data, some mismatch was evident between ADCP data and SSHA data from transect 8. This is likely due to artificial gridding of satellite data and land contamination (i.e., coastal topography echoes^50,51^). In this case, the *in-situ* ADCP data (bottom panels Fig. 2) alone were a better indicator of where the transect was positioned within the ME.

While the effects of eddies on the physical environment are relatively well known^1,45,52^, empirical data on the effects of eddies and eddy edges on zoo-and ichthyoplankton distributions are relatively rare^8^, owing at least partially to the difficulties of sampling non-stationary eddies with net systems. The few high spatial resolution net sampling campaigns investigating eddies and their edges regarding mesozoo-, and ichthyoplankton typically space net stations by several kilometres^53,54^, while the vertical resolution attained with net systems ranges from tens to hundreds of meters. Taken together, such sampling substantially limits the detectability of ecological responses to fronts, convergence, and filaments occurring at eddies^1,18^. Acoustic systems provide high spatial resolution but typically cannot resolve different types of mesozooplankton and ichthyoplankton. Thus, traditionally there has been a mismatch between our ability to sample physical variables at fine spatial and temporal scales, and our ability to sample planktonic biodiversity. Our fine-scale *in-situ* plankton distributions were collected at the scale of the individual and at rates comparable to the collection of physical variables, enabling for the first time, a fine-scale analysis of the distribution of plankton across a mesoscale eddy.

During the lifecycle of an eddy, there usually are three phases: *enrichment*, *concentration*, and *retention* (Ocean Triad configuration^1,17^). During the enrichment phase of a forced cyclonic eddy, nutrients are upwelled, providing fuel for phytoplankton growth. Meanwhile, at the eddy edge, plankton are concentrated due to the convergence of eddy and outside-eddy water masses. When the cyclonic eddy later transitions into its free state, convergence in the eddy interior can concentrate plankton in deeper waters, while the same water then flows outward to the upwelling eddy edge, leading to an aggregation of plankton between the deeper downwelled water and the eddy edge^1,17^. Sailfish are thought to seek out this eddy edge for spawning, since this food-rich environment is favourable for their larvae^28^.

Larval fishes and their copepod prey were concentrated at the edges of the ME we sampled. A known mechanism of plankton accumulation at the edges of such upwelling eddies is the pushing of higher production from the eddy core towards the eddy edge as a result of the divergent flow^55^. In a similar way, *Oithona* and larval fishes likely accumulated at the decaying eddy's edge in this study, as a result of downwelled eddy water. This accumulation may also have been subsidized by zooplankton actively swimming against the downward flow at the edge^56^ to exploit the accumulation of other particles and prey items in this zone. Dense patches of larval fishes spatially overlapped with *Oithona* spp. in the ME's interior, just inward of the eddy edge, on transects 1 and 8. Larval fishes and *Oithona* spp. were also found to overlap spatially in the interface waters of transect 1. Further, a filament of eddy interface water was detected on transect 1, and dense accumulations of *Oithona* and larval fishes were found in and around it. Eddy filaments have been shown to be important for the feeding of top predators such as tuna, squid, seabirds, and cetaceans^19–21^, however quantitative observations are rare^21^, especially for lower trophic levels. While tagging and direct visual observations of some top-predators provide individual-level data for these large consumers, sampling eddy filaments for mesozoo-, and ichthyoplankton is more challenging. By demonstrating elevated concentrations of larval fishes and copepods in association with these filaments, our imagery can further explain the occurrences of top-predators. While the interface water filament on transect 1 likely lead to enhanced submeso-, and microscale turbulences^57,58^, potentially entraining or otherwise attracting *Oithona* and larval fish into the IF water mass, this feature was absent from transect 8 where water masses were more clearly divided.

The small (*~ *600 μm) cyclopoid copepod *Oithona* is a very abundant and important food source in the world's oceans^59^. As a relatively weak swimmer, *Oithona* has been found to be entrained by eddies^60^. Consistent with these observations, *Oithona* in our study were abundant and closely related to variables pertaining to the physical manifestation of the ME (e.g., current speed and direction, water mass). The presence of detritus and diatom concentrations as top predictors in the *Oithona* models is a further indicator of the passive accumulation of *Oithona* at the eddy edge since both detritus and diatoms are passively drifting particles. Physonect siphonophores, albeit rare in our data, were also an important predictor of *Oithona* spp. concentrations. Their distributions largely followed the physical variables of the ME, except in one of the *Oithona* models. Physonects are known to prey on copepods as well as larval fishes^61,62^ and their directional swimming ability^63,64^ may enable them to manoeuvre towards their prey. However, the degree to which they are passively accumulated or actively seek the same *Oithona* regions is unknown. The overlap between *Oithona* and their prey, detritus and diatoms^65^, is likely simply due to passive advection of both to the same areas at the eddy edge.

*Oithona* is a preferred prey of larval fishes^66,67^. Documented families of larval fish that feed on *Oithona* in the Florida Straits include Mullidae, Lutjanidae, Serranidae, and Pomacentridae^66^. While *Oithona* was an important predictor for larval fish concentrations in this study, removing *Oithona* from the larval fish models reduced the explained variances of the models only marginally (0.3% and 2%, on transects 1 and 8, respectively; at over 90% total variance explained). Since all other top predictors in each model reflect the physical footprint of the ME (except for physonect siphonophores in one of the *Oithona* models as noted above), and *Oithona* itself was driven almost exclusively by eddy physics, we hypothesize that larval fishes were also largely passively transported. However, larval fishes are known to orient and swim actively^68–70^, and such active behaviour may have been used to further reduce distances to their *Oithona* prey (as indicated by the added variance explained when including *Oithona* as an explanatory variable), ultimately leading to the very tight, spatial coupling observed. Thus, contrary to our expectations, the distributions of larval fishes in relation to their prey were more influenced by passive transport than active behaviour on the part of the larvae.

Interestingly, the structuring effect of the eddy on planktonic populations did not weaken over the sampled timeframe, and its effects on multiple plankton taxa were still strong just four days before total dissipation. Previous studies have shown that larval fishes entrained in MEs experience enhanced growth and survival^8,9^, and our results demonstrate that this is likely due to overlap in prey and predator concentrations at the eddy edge. Spatial overlap, in this case caused by physical transport, is ultimately needed for successful predator-prey interactions, feeding, and larval growth.

Examining our results in the context of an *Ocean Triad* setting^1,17^, the ME we sampled was in its last life stage, *retention*, as it spun down and lost energy. The fact that the eddy, so close to dissipation, was still sufficiently defined to retain larval fishes sheds new light on the extent to which eddies can shape the pelagic realm, further defining pathways of population connectivity^15^. As ubiquitous features of the ocean, the implementation of eddies, their energy transport, and effects on the plankton into mechanistic models is valuable^71^. Eddy physics and parameterizations are currently included in several models (i.e., ocean circulation, biophysical, climate) to varying degrees, often depending on model resolution^72^. However, eddy spin-down and dissipation are among the least understood pieces of the eddy process, and thus are more difficult to model than other processes (see GEOMETRIC framework for parametrization of MEs in coarse-resolution models^71^). Empirical results such as those from our study could be used to ground-truth models predicting phyto-, and zooplankton distributions affected by MEs, as validation of the underlying coupled physical-biogeochemical models, as well as to add key insights into how biological components may actively interact with one another. Such ground-truthing would arguably only be possible with the high-resolution data collected by underwater imagers in conjunction with deep learning for the analysis of the millions of images, since traditional net sampling would not provide sufficiently resolved data. Although research on underwater imaging began several decades ago^31^, the use of large volumes of imagery to answer ecological questions is in its infancy^73^. Further comparisons of *in situ* imaged plankton distributions across eddies in conjunction with model predictions would further advance our understanding of the role and function of these ubiquitous features in the world's oceans.

## Methods

### Study area

The Florida Current (FC) is a major western boundary current and part of the Gulf Stream system, connecting the Caribbean Sea with the Atlantic Ocean. The FC is strongly influenced by the upstream Loop Current, which comes out of the Gulf of Mexico^52^. Cyclonic mesoscale eddies (MEs) often form in the eastern Gulf of Mexico and the southern Straits of Florida, and propagate along the FC front, skirting the Florida Keys shelf^45,52,74^ (Fig. 1). These MEs have significant effects on the Florida Keys reef system^45^ including enhancing productivity in their centres^4^, enhancing the growth and survival of larval fishes^8,9^, and transporting larval fishes to nearshore reefs^13,14^.

### The *In-situ* Ichthyoplankton Imaging System (ISIIS)

ISIIS^27^ is a towed shadowgraph imager that utilizes a line-scan camera to image a large volume of water (150–185 L^−1^) to intercept relatively rare ichthyoplankton^75^. With a focus on these rare plankton, the volume of water that ISIIS images is orders of magnitude higher than that of other imaging systems (e.g., VPR^32^, LOKI^34,37^). ISIIS's large imaging frame, with a 13 ×13-cm field of view and 50 cm depth of field allows for the undisturbed imaging of a variety of plankton types including fragile gelatinous zooplankton^29,41,42,76^. The resulting images have a pixel resolution of 66 μm. Due to the nature of ISIIS's line-scan camera^27^, the recorded imagery can be interpreted as one large image (*e.g*., a 100 km long image if ISIIS is towed 100 km), with organisms recorded on the scale of the individual. Data are sent to a top-side computer using a fibre optic cable where ISIIS data are time-stamped and additional backups made. ISIIS is equipped with a CTD (Sea-Bird SBE 49 FastCAT), as well as environmental sensors that record dissolved oxygen (Sea-Bird 43), fluorescence (Wet Labs FLRT), and photosynthetically active radiation (PAR; Biospherical QCP-2300).

### Eddy Sampling

During two 2-wk summer cruises in 2014 and 2015 we sampled zoo-, and ichthyoplankton in the Straits of Florida using ISIIS. Onboard the R/V FG *Walton Smith*, ISIIS was towed in an undulating fashion at a ship speed of 2.5 m s^−1^. Data from each cruise (May 28 - June 14, 2014 and June 10-26, 2015; see Supplementary Fig. S5 for all transects) were used in the training and testing of the sparse Convolutional Neural Network (sCNN; see below); however, the adaptive sampling of a mesoscale eddy (ME) reported here occurred from June 10-16, 2015, off the Florida Keys (Fig. 1). This sampling included 13 multi-hour transects where the ISIIS imager was undulating from 3 to 80 m depth (Supplementary Table S5). Distances covered on single transects ranged from 17–70 km.

### Training the sparse Convolutional Neural Network (sCNN) on ISIIS data

The video data obtained by ISIIS were segmented into single frames, and the frames flat-fielded. A k-harmonic means clustering algorithm was used on the flat-fielded frames to detect single regions of interest (ROI; i.e., a single plankton specimen) and these ROIs (hereafter referred to as vignettes) were then saved^42^. Within the 2014 dataset, 124 different categories of plankton (Supplementary Table S6) and particles were identifiable, and 61,571 vignettes representing that diversity were selected to train the sCNN classifier. The sCNN analyses images using multiple layers that are chained together as a network. These layers analyse images in a hierarchical fashion, where the first layer detects very general features in an image (*e.g*., a straight or curved line), and the following layers detect progressively more complex features in the images (*e.g*., the fin of a larval fish). An important aspect of the sCNN is also that layers can exchange information (*i.e*., backpropagation)^42^.

Due to the disproportionally high number of vignettes of common groups, and few images of rare organisms, the number of training vignettes for most classes ranged between several hundreds to thousands of vignettes, while for rare taxa the number of vignettes ranged from 20–100. This situation was ameliorated by the sCNN's ability to augment data which included stretching, rotating and blurring images during the training phase^42^. The sCNN implementation used was *SparseConvNets with Fractional Max‐Pooling*^42,77^. The sCNN was trained until the error rate plateaued at ~ 5%, after 400 epochs.

### Testing the sCNN

138,374 vignettes were randomly extracted from all 2014 and 2015 cruise data and manually identified to generate an unbiased test case. The vignettes were each identified using the trained sCNN, generating a probability that each vignette belonged to any one of the 124 classes (probabilities per vignette sum to one), where the class with the highest probability is selected as the correct automated identification. The 124 original classes were then mapped onto 40 broader groups (e.g., chaetognaths of different shapes into one group; Supplementary Table S6). Probability filtering was applied to separate out vignettes of low classification confidence^42^. Removal of these “low‐confidence images” still allows for the prediction of true spatial distributions^78^. The approach uses a Loess model to determine at which probability threshold a cut-off should be made (at the original class level) to reach 90% classification precision at the broader group level. This is achieved by iteratively removing images of a class below a certain threshold and recalculating classifier precision^42^ (see Supplementary Table S6 for the determined thresholds). Vignettes with a maximum assigned probability less than or equal to the determined thresholds were re-classified as unknown.

To obtain a final classifier performance, a confusion matrix for another independent test set of vignettes was generated (143,418 vignettes from 2014 and 2015 data). These images were identified independently by two human experts, and then via the sCNN. The filtering thresholds (as described above) were applied, mapping the vignettes to their 40 broader groups. The number of true positives (TP), false positives (FP) and false negatives (FN) in the dataset enabled the computation of precision (P = TP/(TP + FP)), recall (R = TP/(TP + FN)), and F1-score (harmonic mean of precision and recall, F1 = 2*P*R/(P + R)). While precision can be interpreted as how many of the selected items were relevant/true positives, recall can be interpreted as the number of relevant items that were selected. Since the F1-score takes into account precision as well as recall, it is the preferred metric to gauge the ability of a classifier to predict a class.

### Automated identifications using the sCNN and post-processing of data

Once vignettes collected during the sampling of the eddy (June 10-16, 2015) were identified using the sCNN, and vignettes with a low confidence classification removed, identifications were merged with the environmental data collected by ISIIS, and binned into 1-m vertical strata. The resulting data were used to estimate concentrations of plankton (ind. m^−3^) and particles based on the volume of water imaged by ISIIS in each 1-m vertical stratum. A correction factor was applied to these concentrations based on confusion matrix results (Correction factor(taxon) = Precision(taxon)/Recall(taxon)). Using this correction factor approach was shown to reproduce concentrations from expert counts^38^. ISIIS-derived organismal and environmental data for each of the 13 eddy transects (Fig. 1) were then kriged (R package ‘gstat’^79^) onto a grid spanning the length of each transect, at 1-m vertical and 500-m horizontal resolution.

### Environmental and ecological data analyses

#### Identification of different water masses

ADCP data collected by the R/V FG Walton Smith (Teledyne RD Instruments; 600 kHz Workhorse Mariner and 75 kHz Ocean Surveyor) during ISIIS transects were analysed and used to determine the geographic position of the eddy. Based on the zonal (*u*)- and meridional (*v*) vectors, the resulting direction and speed of the current were calculated using the uv2ds function of the R package ‘rWind’^80^. Magnitudes of *u* and *v*, as well as the resulting speed of the current were then used in k-means unsupervised clustering^81^, a proven approach to distinguishing water masses^82^. The optimal number of significant clusters was determined using the ‘Total within sum of squares’ measure. To apply the k-means model to the whole transects, the *u*-, and *v* vectors were kriged the same way as the organismal data, and from these vectors the speed of the current was again calculated using the uv2ds function of the ‘rWind’ R-package.

To investigate the effect of the different water masses on plankton concentrations, ANOVAs were used to compare the distributions of different taxa by water mass. For taxa and transect combinations where significant differences were found, Tukey HSD tests were used to identify between which water masses taxa concentrations differed significantly. As part of the k-means analysis, Florida Current (FC) water was identified as one cluster. As a potentially important boundary for plankters, the distance from each point on the transect kriging grid to the FC water mass was calculated using the gDistance function of the R package ‘rgeos’^83^.

Daily satellite-derived sea surface height anomaly (SSHA) information^84^ was used to place the *in-situ* data collected by ISIIS and ship-born ADCP into geographic perspective. To aid with the localization of eddy-induced up-, and downwelling, mixed layer depth along the transects^85^, as well as temperature and salinity anomalies, were calculated.

#### Random Forests analyses

Kriged organismal data from ISIIS were merged with kriged chlorophyll *a*, dissolved oxygen, and density data, as well as the distance-to-FC-water variable, the categorical variable water mass as derived from the k-means clustering, and current speed and direction from the ADCP. This merged dataset (Table 1) was then used in multiple Random Forests models^86,87^, to predict the concentrations of larval fishes. This type of ecological niche model is a powerful tool for determining which environmental drivers best describe a taxon's ecological niche^88^. For each Random Forests model 500 trees were grown, while each tree was grown from a random subset of 14 predictors. Using the same Random Forests settings ensured comparability of the results.

The importance of predictors in the Random Forests models was ranked based on their effects on node purity (an indicator for how well nodes in the decision trees split the data, based on a loess function of the mean squared error), and their reduction of the mean squared error of the model using the R-package ‘randomForestExplainer’^89^. The detailed effects of the five most important predictors in the Random Forests models were further investigated using Accumulated Local Effects (ALE) plots using the ‘iml’ R-package^90,91^. ALE plots visualize the effect of the full range of an explanatory variable (continuous and categorical) on the mean model prediction, and are an unbiased alternative to partial dependence plots, providing a better accounting of correlated explanatory variables.

## Supplementary information

Supplementary Information.

## Data Availability

Please visit the Biological and Chemical Oceanography Data Management Office (BCO-DMO) at https://www.bco-dmo.org/project/528606 for ISIIS data as well as other cruise data (e.g., from CTDs).
