# Supplementary material for: Prey and predator overlap at the edge of a mesoscale eddy: fine-scale, in-situ distributions to inform our understanding of oceanographic processes
Source: Sci Rep. 2020 Jan 22;10:921. doi: 10.1038/s41598-020-57879-x (PMC6976709; doi:10.1038/s41598-020-57879-x)
Supplement: Supplementary file 1 — Supplementary Information. [file 41598_2020_57879_MOESM1_ESM.pdf]

Supplementary material

Prey and predator overlap at the edge of a mesoscale eddy: fine-scale, in-situ distributions to inform our understanding of oceanographic processes

Moritz S Schmid[1*], Robert K Cowen[1], Kelly Robinson[2], Jessica Y Luo[3], Christian Briseño-Avena[1,4], Su Sponaugle[5]

[1] Hatfield Marine Science Center, Oregon State University, Newport, OR 97365, USA
[2] Department of Biology, University of Louisiana at Lafayette, Lafayette, LA 70503, USA
[3] NOAA Geophysical Fluid Dynamics Laboratory, Princeton, NJ 08540, USA
[4]Department of Environmental and Ocean Sciences, University of San Diego, San Diego, CA 92122, USA
[5] Department of Integrative Biology, Hatfield Marine Science Center, Oregon State University, Newport, OR 97365, USA.
*Corresponding author: schmidm@oregonstate.edu

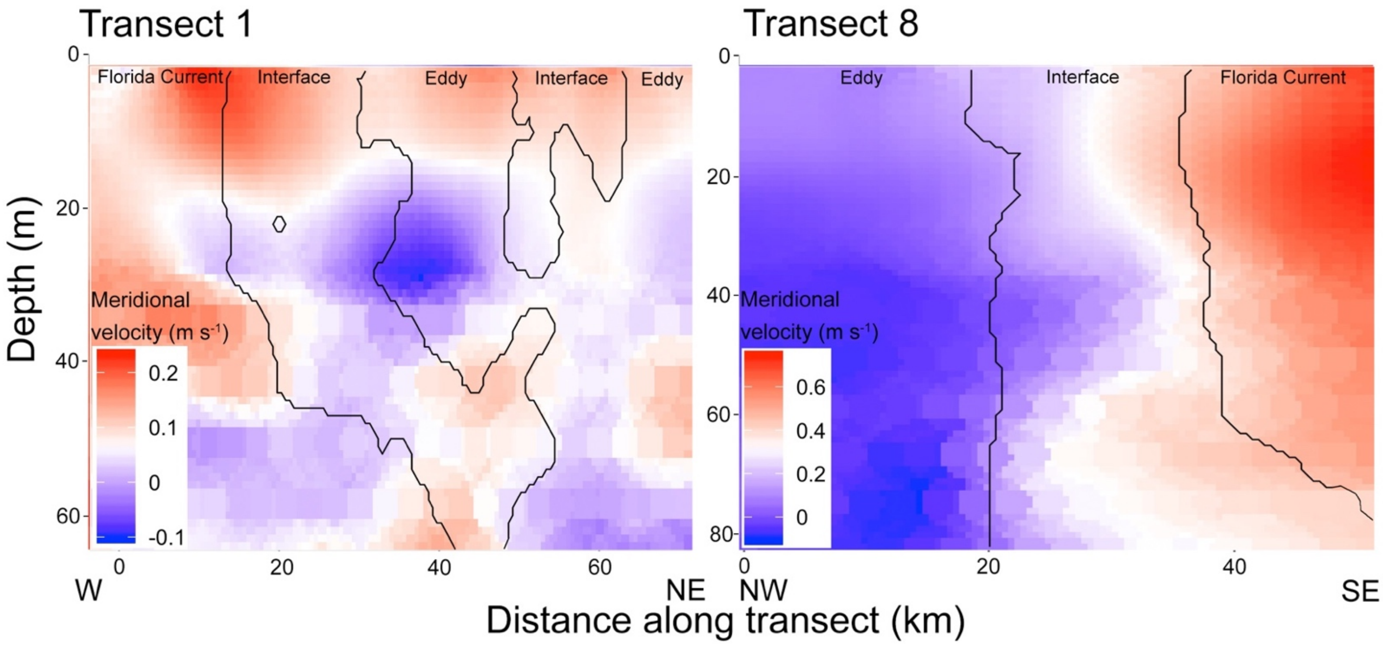

Supplementary Figure S1: Meridional velocities encountered along transects 1 and 8. For both transects, distance along transect begins at the westernmost end of the transect. Overlaid on top of the velocities are the contours of the water masses as quantified by k-means clustering (see Methods). Transect 1 was sampled on 6/10/2015, and transect 8 on 6/14/2015.

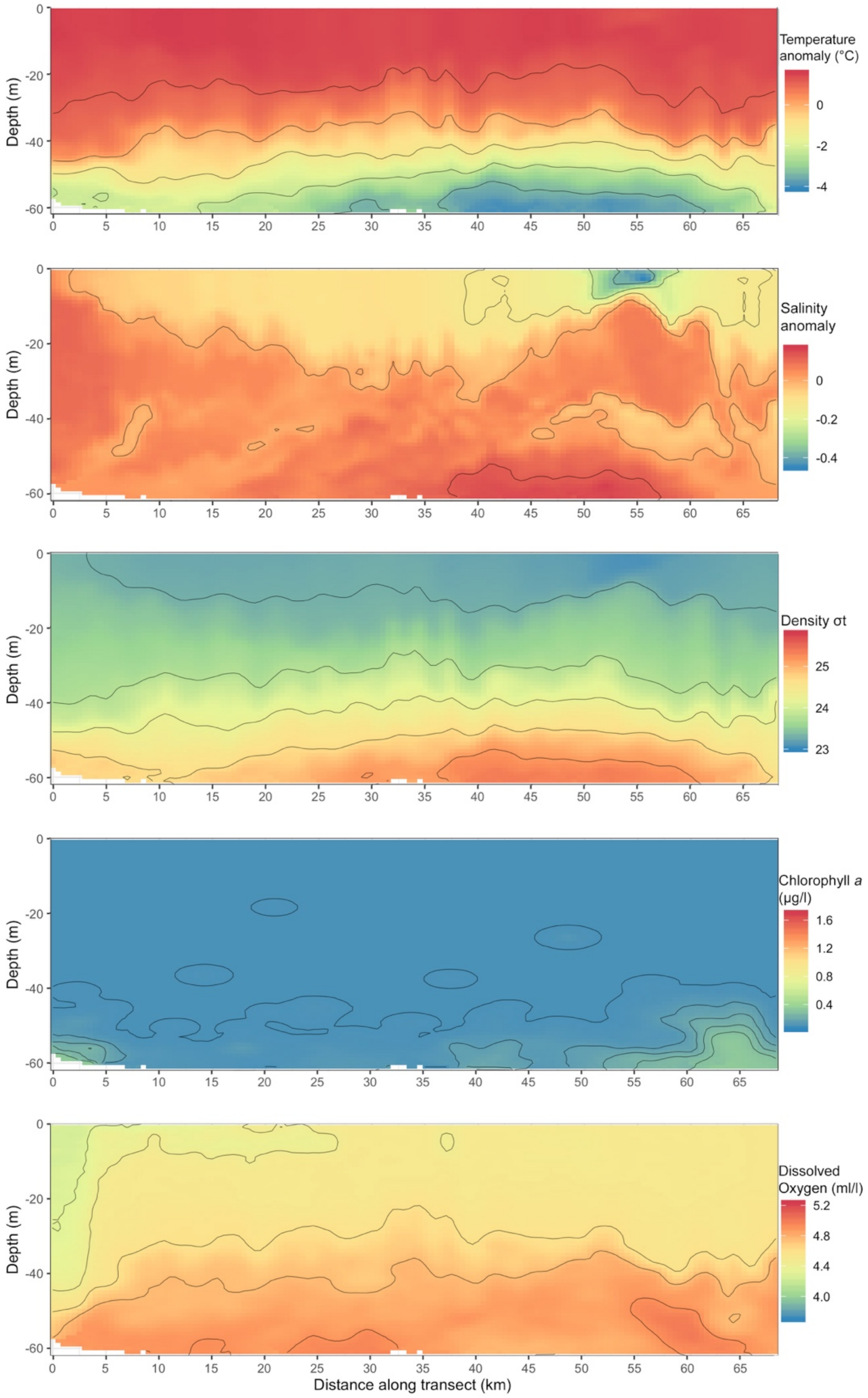

Supplementary Figure S2: Environmental data recorded by ISIIS on transect 1. Temperature and salinity are given as anomalies from the mean. Distance along the transect starts in the West and ends in the Northeast.

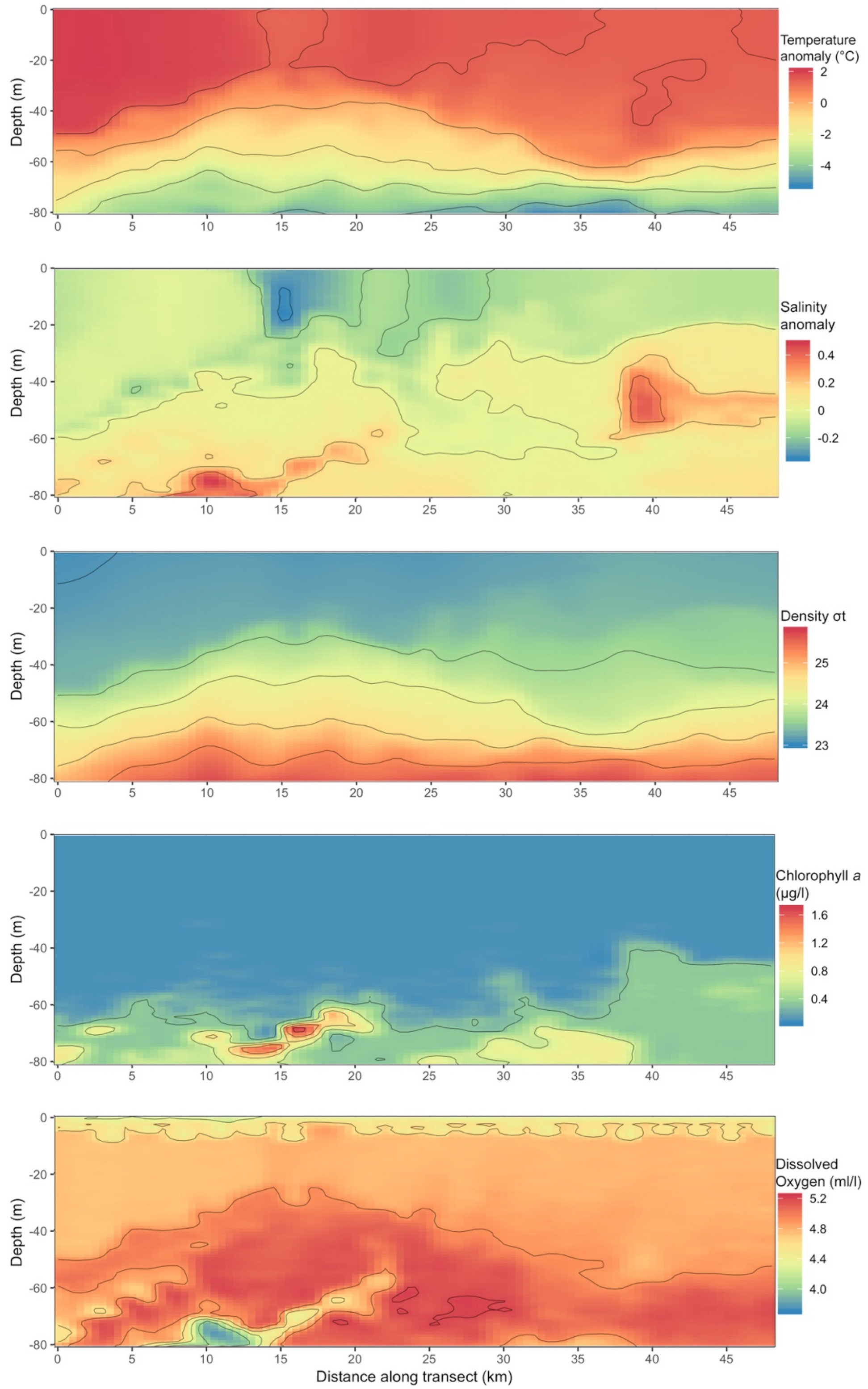

Supplementary Figure S3: Environmental data recorded by ISIIS on transect 8. Temperature and salinity are given as anomalies from the mean. Distance along the transect starts in the Northwest and ends in the Southeast.

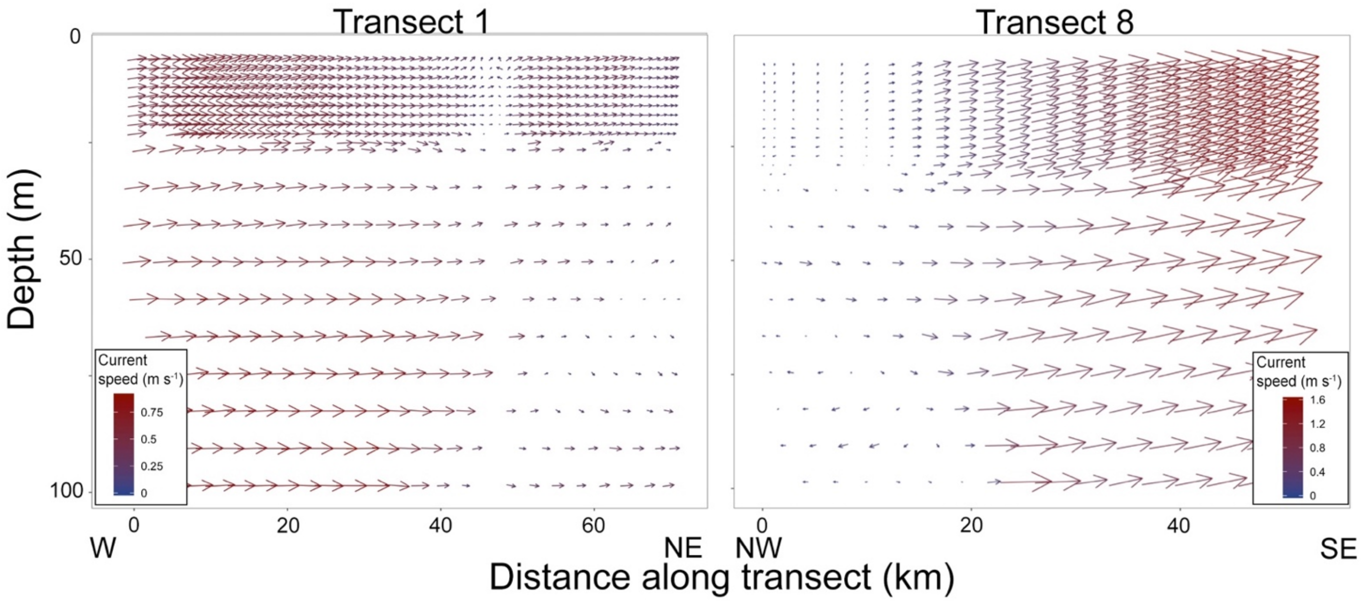

Supplementary Figure S4: Quiver plots indicating current direction and speed along transects 1 and 8 (Cartesian coordinates). For both transects, distance along transect begins at the westernmost end of the transect. Short arrows indicate the slower eddy within the faster eastward flowing Florida Current.

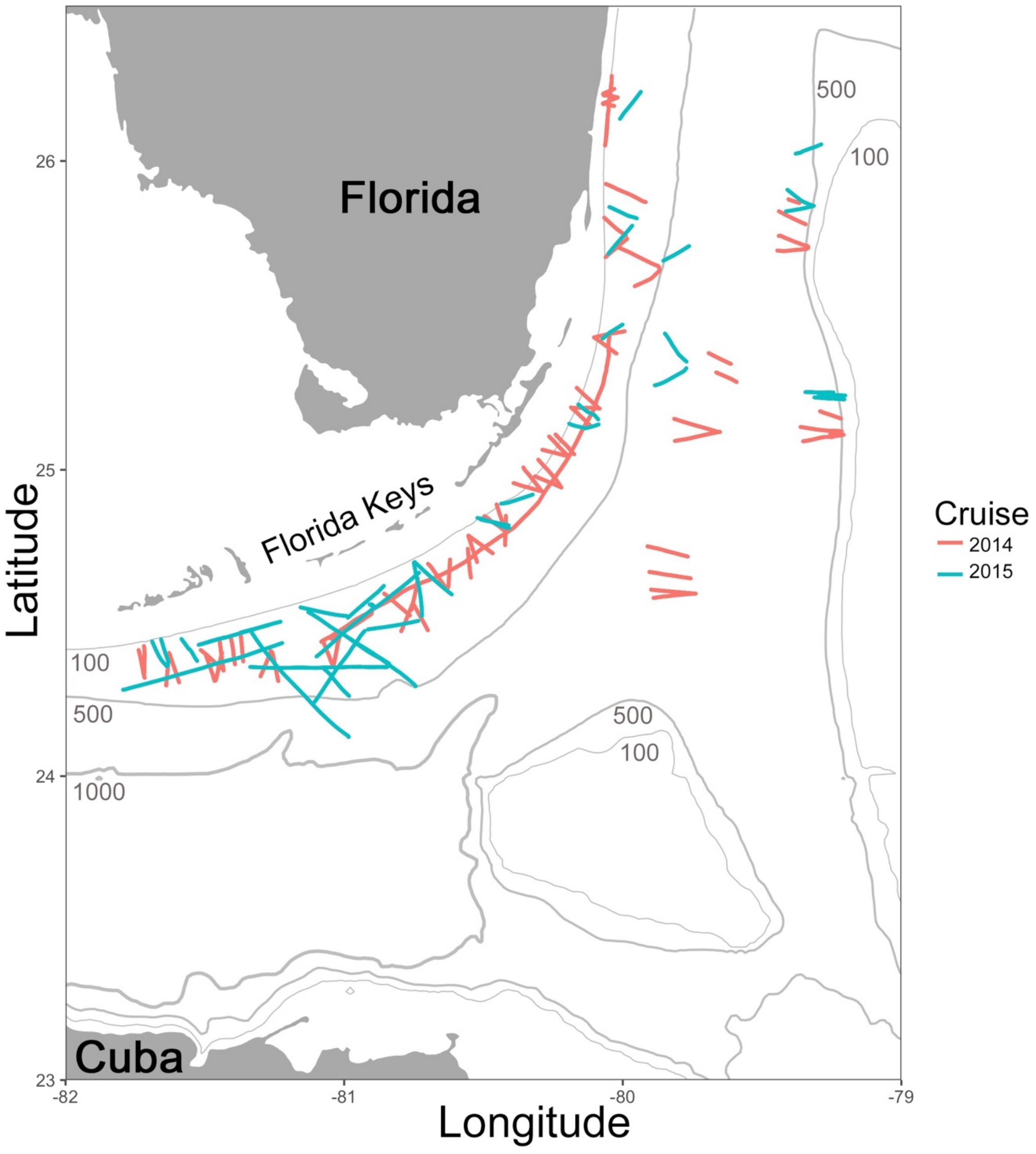

Supplementary Figure S5: All ISIIS transects sampled during 2014 (delineated in red) and 2015 (delineated in blue). While the sparse convolutional neural network (sCNN), that was used to identify species on images in an automated fashion was trained on data from the 2014 cruise, it was tested on data from 2014 and 2015. Testing on both years ensured that the sCNN classifier generalized well. This figure was made in R[81].

Supplementary Table S1: K-means signatures of the three significant clusters delineating the different water masses on transect 1 (T1) and transect 8 (T8). U and V components (m s^{-1}) as well as resulting current velocities (m s^{-1}, Cartesian) are the means of their respective clusters.

Water mass	Transect	U component (m s^{-1})	V component (m s^{-1})	Current Velocity (m s^{-1})
Florida Current	T1	0.64	0.06	0.64
Interface	T1	0.40	0.05	0.41
Eddy	T1	0.21	0.04	0.23
Florida Current	T8	1.22	0.62	1.37
Interface	T8	0.65	0.26	0.70
Eddy	T8	0.10	0.02	0.15

Supplementary Table S2. Confusion matrix measures of the sCNN; including precision, recall and F1 (harmonic mean of precision and recall) metric for the 40 groups found along the transects.

Group	True positives	False positives	False negatives	Total	Precision	Recall	F1	Correction factor
acantharia_protist	578	33	483	1061	94.6	54.5	69.2	1.74
appendicularian	4819	192	418	5237	96.2	92	94.1	1.05
artifact	25397	97	266	25663	99.6	99	99.3	1.01
chaetognath	1394	91	164	1558	93.9	89.5	91.6	1.05
copepod_calanoid	1927	56	477	2404	97.2	80.2	87.9	1.21
copepod_copilia	2	0	9	11	100	18.2	30.8	5.49
copepod_oithona	974	21	241	1215	97.9	80.2	88.2	1.22
copepod_oithona_eggs	403	25	35	438	94.2	92	93.1	1.02
copepod_other	760	37	51	811	95.4	93.7	94.5	1.02
crustacean_other	10	3	93	103	76.9	9.7	17.2	7.93
ctenophore_cestid	2	0	11	13	100	15.4	26.7	6.49
ctenophore_cydippid	6	1	41	47	85.7	12.8	22.3	6.70
ctenophore_lobate	1	0	9	10	100	10	18.2	10.00
detritus	47926	1905	1228	49154	96.2	97.5	96.8	0.99
diatom	20020	1227	911	20931	94.2	95.6	94.9	0.99
doliolid	34	0	431	465	100	7.3	13.6	13.70
echinoderm_other	3	1	45	48	75	6.2	11.5	12.10
echinoderm_seastar	134	14	219	353	90.5	38	53.5	2.38
echinoderm_urchin	176	28	218	394	86.3	44.7	58.9	1.93
fecal_pellet	15142	350	872	16014	97.7	94.6	96.1	1.03
fish_larvae	75	10	107	182	88.2	41.2	56.2	2.14
heteropod	0	0	4	4	NA	0	NA	NA
hydromedusae	332	34	304	636	90.7	52.2	66.3	1.74
hydromedusae_narco	453	79	115	568	85.2	79.8	82.4	1.07
jellyfish_other	42	14	248	290	75	14.5	24.3	5.17
pelagic_tunicate	19	0	67	86	100	22.1	36.2	4.52
polychaete	35	24	37	72	59.3	48.6	53.4	1.22
protist	2045	232	2509	4554	89.8	44.9	59.9	2.00
pteropod	13	0	64	77	100	16.9	28.9	5.92

radiolarian	103	5	104	207	95.4	49.8	65.4	1.92
salp	20	4	33	53	83.3	37.7	51.9	2.21
shrimp_decapods	46	5	44	90	90.2	51.1	65.2	1.77
shrimp_euphausiids	17	1	37	54	94.4	31.5	47.2	3.00
shrimp_other	5	1	19	24	83.3	20.8	33.3	4.00
siphonophore_calycophoran	199	4	287	486	98	40.9	57.7	2.40
siphonophore_other	5	1	12	17	83.3	29.4	43.5	2.83
siphonophore_physonect	33	7	19	52	82.5	63.5	71.8	1.30
tornaria	13	1	0	13	92.9	100	96.3	0.93
trichodesmium	5657	56	2794	8451	99	66.9	79.8	1.48
unknown	754	9006	539	1293	7.7	58.3	13.6	0.13
**Weighted mean**	-	-	-	-	**95.6**	**90.5**	**93**	-

Supplementary Table S3: Average plankton concentrations (# m^{-3}) on transects 1 and 8. * indicates concentrations below 0.1 m^{-3}.

Taxa	Average concentration on transect 1	Average concentration on transect 8
detritus	1347.5	959.4
diatom	248.9	263.4
fecal_pellet	206.9	189
trichodesmium	140	85.3
appendicularian	74.6	69.8
copepod_oithona	60.8	15
protist	45.3	36.9
copepod_calanoid	26.9	31.3
chaetognath	21	18.8
copepod_other	13	13.9
hydromedusae_narco	7.4	12
fish_larvae	5.6	2.3
acantharia_protist	4.9	6
hydromedusae	3.9	3.8
copepod_oithona_eggs	3.7	3.5
echinoderm_urchin	2.6	3.7
echinoderm_seastar	2.2	1.4
siphonophore_calycophoran	1.8	1.1
radiolarian	1.4	1.1
shrimp_euphausiids	0.7	0.7
jellyfish_other	0.7	0.7
polychaete	0.3	0.4
shrimp_decapods	0.3	0.8
pteropod	0.2	0.3
doliolid	0.2	0.3
crustacean_other	0.2	0.3
siphonophore_physonect	0.1	0.2
ctenophore_cestid	0.1	0.1
pelagic_tunicate	0.1	0.2
siphonophore_other	0.1	*

salp	0.1	0.1
ctenophore_cydippid	*	0.1
shrimp_other	*	0.1
copepod_copilia	*	0.1
heteropod	*	*
ctenophore_lobate	*	*
tornaria	*	0.1

Supplementary Table S4: Tukey HSD tests of the effects of the different water masses on larval fish and *Oithona* spp. concentrations. ED = Eddy water, FC = Florida Current water, IF = Interface water, CI = Confidence Interval, ns = not significant.

**Transect**	**Taxon**	**Tukey HSD combinations**	**lower CI**	**upper CI**	**p-value**
1	*Oithona* spp.	ED - FC	40	44.7	***
1	*Oithona* spp.	ED - IF	16.7	21.4	***
1	*Oithona* spp.	IF - FC	20.1	25.7	***
1	Fish larvae	ED - FC	4.9	5.4	***
1	Fish larvae	ED - IF	2.2	2.6	***
1	Fish larvae	IF - FC	2.5	3	***
8	*Oithona* spp.	ED - FC	19.2	20.9	***
8	*Oithona* spp.	ED - IF	17.3	18.6	***
8	*Oithona* spp.	IF - FC	1.2	2.9	***
8	Fish larvae	ED - FC	2.4	2.6	***
8	Fish larvae	ED - IF	2.3	2.5	***
8	Fish larvae	IF - FC	-0.01	0.2	ns

*** $p < 0.0001$

Supplementary Table S5. Transects sampled for the eddy study. Maximum sampling depth is given as the deepest depth observed during the undulating transect. Distance sampled is given as the distance from start lat/lon to end lat/lon, not along the flight path (undulations) of the ISIIS vehicle.

Transect	Start date and time (EDT)	End date and time (EDT)	Start lat / lon (degrees)	End lat / lon (degrees)	Max sample Depth (m)	Distance sampled (km)
1	6/10/15 21:25	6/11/15 06:00	24.44/-81.22	24.34/-81.69	72.79	69.5
2	6/11/15 08:58	6/11/15 18:39	24.43/-81.52	24.51/-81.23	66.37	49.0
3	6/12/15 12:36	6/12/15 18:11	24.47/-81.33	24.13/-80.98	86.53	52.4
4	6/13/15 09:18	6/13/15 12:35	24.26/-80.98	24.35/-81.08	65.42	14.0
5	6/13/15 14:00	6/13/15 19:54	24.39/-81.10	24.67/-80.73	92.31	52.9
6	6/14/15 07:20	6/14/15 09:03	24.62/-80.86	24.52/-80.99	64.57	17.5
7	6/14/15 19:01	6/14/15 20:40	24.52/-80.99	24.55/-81.16	85.55	17.2
8	6/14/15 21:41	6/15/15 03:06	24.54/-81.13	24.29/-80.74	81.91	47.9
9	6/16/15 00:28	6/16/15 08:17	24.36/-80.85	24.36/-81.34	85.60	50.3
10	6/16/15 11:24	6/16/15 14:37	24.24/-81.11	24.47/-80.93	82.52	31.6
11	6/16/15 14:46	6/16/15 16:36	24.48/-80.92	24.51/-80.73	81.49	18.7
12	6/16/15 16:48	6/16/15 18:55	24.52/-80.72	24.70/-80.74	82.72	20.7
13	6/16/15 18:56	6/16/15 20:38	24.69/-80.74	24.59/-80.61	82.04	17.1

Supplementary Table S6. The original 124 sCNN classes, their respective filtering thresholds, and the 40 broader groupings the original classes were mapped to for the ecological analyses. The development of the filtering threshold, which generally leads to the exclusion of low-confidence images, is described in detail in the methods section.

**All classes in sCNN**	**Filter probability**	**Groups for ecological analyses**
acantharia_protist	0.94	acantharia_protist
acantharia_protist_big_center	0.35	acantharia_protist
acantharia_protist_halo	0.34	acantharia_protist
appendicularian_fritillaridae	0.29	appendicularian
appendicularian_s_shape	0.28	appendicularian
appendicularian_slight_curve	0.46	appendicularian
appendicularian_straight	0.35	appendicularian

artifacts	0.24	artifact
artifacts_edge	0.67	artifact
chaetoganth_curve	0.51	chaetognath
chaetognath_s	0.32	chaetognath
chaetognath_straight	0.14	chaetognath
chaetognath_tail	0.34	chaetognath
chateognath_head	0.84	chaetognath
copepod_calanoid_calocalanus	0.27	copepod_calanoid
copepod_calanoid_eggs	0.16	copepod_calanoid
copepod_calanoid_eucalanus	0.64	copepod_calanoid
copepod_calanoid_flatheads	0.25	copepod_calanoid
copepod_calanoid_frillyantennae	0.26	copepod_calanoid
copepod_calanoid_longantennae	0.25	copepod_calanoid
copepod_calanoid_small_oval	0.21	copepod_calanoid
copepod_calanoid_typeA	0.22	copepod_calanoid
copepod_calanoid_typeB	0.26	copepod_calanoid
copepod_calanoid_typeC	0.20	copepod_calanoid
copepod_cyclopoid_copilia	0.92	copepod_copilia
copepod_cyclopoid_oithona	0.66	copepod_oithona
copepod_cyclopoid_oithona_eggs	0.26	copepod_oithona_w_eggs
copepod_sideview	0.21	copepod_other
crustacean_amphipod_large	0.67	crustacean_other
crustacean_amphipod_thin	0.78	crustacean_other
crustacean_other	0.85	crustacean_other
crustacean_stomatopod	1.00	crustacean_other
crustacean_zoea	0.73	crustacean_other
ctenophore_cestid	0.93	ctenophore_cestid
ctenophore_cydippid_no_tentacles	0.84	ctenophore_cydippid
ctenophore_cydippid_tentacles	0.86	ctenophore_cydippid
ctenophore_lobate	0.95	ctenophore_lobate
detritus	0.26	detritus
diatom_chain_string	0.32	diatom
diatom_chain_tube	0.37	diatom
echinoderm_auricularia_larva	1.00	echinoderm_other
echinoderm_juvenile_brittlestar	0.64	echinoderm_seastar
echinoderm_larva_heart_urchin	0.42	echinoderm_urchin
echinoderm_larva_heart_urchin_irreg	0.54	echinoderm_urchin
echinoderm_larva_pluteus	0.62	echinoderm_urchin
echinoderm_larva_pluteus_early	0.99	echinoderm_urchin
echinoderm_larva_seastar_bipinnaria	1.00	echinoderm_seastar
echinoderm_larva_seastar_brachiolaria	0.22	echinoderm_seastar
echinoderm_larva_typeA	0.89	echinoderm_other
fecal_pellet	0.28	fecal_pellet
fish_clupeid_like_shape	0.33	fish
fish_other	0.34	fish
fish_medium_deep_body	0.93	fish
fish_myctophids	0.88	fish
fish_sm_dorsal_spinesrays	0.89	fish
fish_sm_pointedsnout	0.28	fish

fish_thin_body	0.91	fish
heteropod	0.52	heteropod
hydromedusae_aglaura	0.15	hydromedusae
hydromedusae_bell_and_tentacles	0.27	hydromedusae
hydromedusae_h15	0.70	hydromedusae
hydromedusae_haliscera	0.22	hydromedusae
hydromedusae_liriope	0.23	hydromedusae
hydromedusae_narco_cunina	0.57	hydromedusae_narco
hydromedusae_narco_dark	0.82	hydromedusae_narco
hydromedusae_narco_young	0.55	hydromedusae_narco
hydromedusae_narcomedusae	0.45	hydromedusae_narco
hydromedusae_other	0.76	hydromedusae
hydromedusae_partial_dark	0.97	hydromedusae
hydromedusae_sideview_big	0.24	hydromedusae
hydromedusae_solmaris	0.17	hydromedusae_narco
hydromedusae_solmundella	0.69	hydromedusae_narco
hydromedusae_typeA	0.21	hydromedusae
hydromedusae_typeB	0.52	hydromedusae
hydromedusae_typeC	0.75	hydromedusae
hydromedusae_typeD	0.17	hydromedusae
hydromedusae_typeD_bell_and_tentacles	0.25	hydromedusae
hydromedusae_typeE	0.62	hydromedusae
hydromedusae_typeF	0.94	hydromedusae
jellyfish_ephyra	0.72	jellyfish_other
jellyfish_tentacles	1.00	jellyfish_other
polychaete	0.65	polychaete
polychaete_nechtochaete_magelona	0.50	polychaete
polychaete_trochophore_larvae	0.85	polychaete
protist_dark_center	0.27	protist
protist_dark_circle_2rays	0.28	protist
protist_fuzzy_olive	0.54	protist
protist_noctiluca	0.23	protist
protist_noctiluca_long_flagella	0.43	protist
protist_other	0.99	protist
protist_star	0.37	protist
pteropod_butterfly	0.98	pteropod
pteropod_tail	0.96	pteropod
pteropod_theco_dev_seq	0.54	pteropod
pteropod_triangle	0.98	pteropod
radiolarian_chain	0.84	radiolarian
radiolarian_colony	0.97	radiolarian
shrimp_caridean	0.98	shrimp_decapods
shrimp_escape	0.91	shrimp_other
shrimp_euphausiids	0.88	shrimp_euphausiids
shrimp_lucifer	0.55	shrimp_decapods
shrimp_sergestidae	0.62	shrimp_decapods
shrimp_sm_other	0.63	shrimp_other
siphonophore_calycophoran_abylidae	0.96	siphonophore_calycophoran
siphonophore_calycophoran_rocketship	0.96	siphonophore_calycophoran

siphonophore_calycophoran_sphaeronectes	1.00	siphonophore_calycophoran
siphonophore_calycophoran_young	0.89	siphonophore_calycophoran
siphonophore_partial	0.63	siphonophore_other
siphonophore_physonect	0.56	siphonophore_physonect
siphonophore_physonect_young	0.81	siphonophore_physonect
tornaria_acorn_worm_larvae	0.45	tornaria
trichodesmium_bowtie	0.99	trichodesmium
trichodesmium_multiple	0.34	trichodesmium
trichodesmium_partial	0.75	trichodesmium
trichodesmium_puff	0.57	trichodesmium
trichodesmium_tuft	0.85	trichodesmium
tunicate_doliolid	1.00	doliolod
tunicate_partial	1.00	pelagic_tunicate
tunicate_salp	1.00	salp
tunicate_salp_chains	0.27	salp
unknown_blobs_and_smudges	NA	unknown
unknown_dark_circles	NA	unknown
unknown_sticks	NA	unknown
unknown_unclassified	NA	unknown
